# Unveiling the Performance of Co-Assembled Hybrid Nanocarriers: Moving towards the Formation of a Multifunctional Lipid/Random Copolymer Nanoplatform

**DOI:** 10.3390/pharmaceutics16091204

**Published:** 2024-09-13

**Authors:** Efstathia Triantafyllopoulou, Diego Romano Perinelli, Aleksander Forys, Pavlos Pantelis, Vassilis G. Gorgoulis, Nefeli Lagopati, Barbara Trzebicka, Giulia Bonacucina, Georgia Valsami, Natassa Pippa, Stergios Pispas

**Affiliations:** 1 Section of Pharmaceutical Technology, Department of Pharmacy, School of Health Sciences, National and Kapodistrian University of Athens (NKUA), Panepistimioupolis Zografou, 15771 Athens, Greece; efstrian@pharm.uoa.gr (E.T.); valsami@pharm.uoa.gr (G.V.); 2 School of Pharmacy, University of Camerino, Via Gentile III da Varano, 62032 Camerino, Italy; diego.perinelli@unicam.it (D.R.P.); giulia.bonacucina@unicam.it (G.B.); 3 Centre of Polymer and Carbon Materials, Polish Academy of Sciences, 41-819 Zabrze, Poland; aforys@cmpw-pan.pl (A.F.); btrzebicka@cmpw-pan.pl (B.T.); 4 Molecular Carcinogenesis Group, Department of Histology and Embryology, Medical School, National and Kapodistrian University of Athens (NKUA), 11527 Athens, Greece; pavlospp@biol.uoa.gr (P.P.); vgorg@med.uoa.gr (V.G.G.); 5 Biomedical Research Foundation, Academy of Athens, 11527 Athens, Greece; nlagopati@med.uoa.gr; 6 Ninewells Hospital and Medical School, University of Dundee, Dundee DD1 9SY, UK; 7 Faculty Institute for Cancer Sciences, Manchester Academic Health Sciences Centre, University of Manchester, Manchester M20 4GJ, UK; 8 Faculty of Health and Medical Sciences, University of Surrey, Surrey GU2 7YH, UK; 9 Laboratory of Biology, Department of Basic Medical Sciences, Medical School, National and Kapodistrian University of Athens (NKUA), 11527 Athens, Greece; 10 Theoretical and Physical Chemistry Institute, National Hellenic Research Foundation, 48 Vassileos Constantinou Avenue, 11635 Athens, Greece

**Keywords:** hybrid nanoparticles, drug delivery, lipid, random copolymer, Laurdan probe, cryo-TEM, microcalorimetry, methotrexate

## Abstract

Despite the appealing properties of random copolymers, the use of these biomaterials in association with phospholipids is still limited, as several aspects of their performance have not been investigated. The aim of this work is the formulation of lipid/random copolymer platforms and the comprehensive study of their features by multiple advanced characterization techniques. Both biomaterials are amphiphilic, including two phospholipids (1,2-dioctadecanoyl-sn-glycero-3-phosphocholine (DSPC), 1,2-dioleoyl-sn-glycero-3-phosphocholine (DOPC)) and a statistical copolymer of oligo (ethylene glycol) methyl ether methacrylate (OEGMA) and 2-(diisopropylamino) ethyl methacrylate (DIPAEMA). We examined the design parameters, including the lipid composition, the % comonomer ratio, and the lipid-to-polymer ratio that could be critical for their behavior. The structures were also probed in different conditions. To the best of the authors’ knowledge, this is the first time that P(OEGMA-co-DIPAEMA)/lipid hybrid colloidal dispersions have been investigated from a membrane mechanics, biophysical, and morphological perspective. Among other parameters, the copolymer architecture and the hydrophilic to hydrophobic balance are deemed fundamental parameters for the biomaterial co-assembly, having an impact on the membrane’s fluidity, morphology, and thermodynamics. Exploiting their unique characteristics, the most promising candidates were utilized for methotrexate (MTX) loading to explore their encapsulation capability and potential antitumor efficacy in vitro in various cell lines.

## 1. Introduction

Methotrexate (MTX) is a versatile chemotherapeutic, immunosuppressant, and antineoplastic active pharmaceutical ingredient (API) in clinical use for the treatment of malignancies and inflammatory diseases [1]. The low water solubility, poor bioavailability, and systematic toxicity (i.e., nephrotoxicity) of MTX are the main challenges for formulation scientists in applying it for clinical use [2]. Several publications have appeared in the literature showing the amelioration of the therapeutic index of MTX by its encapsulation into several types of nanoparticles, including micelles, dendrimers, nanocapsules, polymersomes, etc. [3,4,5,6]. The improved solubility in aqueous media, the controlled release, the long-term stability, and the enhanced pharmacokinetic profile have already been achieved by using MTX nanoformulations.

Lipid/polymer hybrid nanosystems have appeared in the literature in recent years as advanced therapeutic delivery systems due to the multitude of their advantages, such as the synergistic properties of their functional materials, physicochemical and morphological versatility, improved loading capacity, biocompatibility, controlled release profiles, and increased colloidal stability [7,8,9,10,11,12,13,14,15,16,17]. On the other hand, the complexity of the formulation, the scale-up challenges, and the regulatory hurdles remain the main obstacles to the clinical translation of these multifunctional drug delivery platforms [14,17,18,19,20,21]. Their unique properties were found to be important for a custom-tailored next-generation approach to cancer therapeutics [22]. The prolonged circulation in the blood or plasma and the selective accumulation in the tumor tissue due to the enhanced permeability and retention effect (EPR), accompanied by the burst release of the anticancer agent in its acidic environment, are ideal properties for an effective drug delivery platform for site-specific targeting of the tumor area. For both biological phenomena, the nanoscale size, the surface functionalization, and the biodegradability and/or fusogenic properties of lipid/polymer hybrid nanosystems are responsible and can be controlled during their design and development processes [22].

MTX was also encapsulated in lipid/polymer hybrid nanoparticles composed of poly(lactic-co-glycolic acid) (PLGA) and the natural phospholipid lipoid S. The high encapsulation efficiency (%EE)—higher than 75%—and the controlled release of MTX from nanosized spherical particles, which showed exceptional colloidal stability and increased cellular uptake due to the lipid coating, made these hybrid nanoparticles ideal for the delivery of this anticancer agent [23]. Additionally, MTX and beta-carotene-loaded lipid polymer hybrid nanoparticles were found to be effective in breast cancer, and beta-carotene exhibited a protective role in the cytotoxicity induced by the chemotherapeutic agent [24].

Lipid/polymer hybrid nanoparticles were fabricated by natural and PEGylated phospholipids, polycaprolactone (PCL), and the co-delivery of MTX and Aceclofenac. These systems exhibited high loading capacity for both the active pharmaceutical ingredients and extremely rapid cell internalization within two hours. The last property played a key role in the effectiveness of the formulation for breast cancer, with improved pharmacokinetics due to the unique characteristics and surface modification of the hybrid platform [25].

pH-responsive hybrid lipid/polymer nanoparticles were prepared by the self-assembled nanoprecipitation method for the loading of docetaxel and its specific cytosolic delivery, resulting in better tumor targeting and accumulation capabilities due to the physicochemical properties of the delivery system. Namely, the biodegradability of the PLGA core and fusogenic properties of 1,2-dioleoyl-sn-glycero-3-phosphoethanolamine (DOPE) caused a synergistic effect with added value for the targeting and site-specific release of the encapsulated anticancer API [26]. pH-sensitive hybrid nanoplatforms are very promising candidates for pharmaceutical applications, especially in cancer therapy due to their asset in spatiotemporal release. Namely, the pH differentiations in vivo enable pH-responsive nanoparticles—compared with conventional ones—to deliver their cargo in a controlled manner at targeted tissues, such as tumors that exhibit different environmental conditions than normal tissues. 

The creation of lipid/random copolymer platforms and a thorough investigation of their characteristics using a variety of physicochemical, thermotropic, and morphological characterization methods are the main goals of this investigation. A statistical copolymer of oligo (ethylene glycol) methyl ether methacrylate (OEGMA) and 2-(diisopropylamino) ethyl methacrylate (DIPAEMA) and two phospholipids—1,2-dioctadecanoyl-sn-glycero-3-phosphocholine (DSPC) and 1,2-dioleoyl-sn-glycero-3-phosphocholine (DOPC)—were used; these biomaterials are amphiphilic, with the copolymers also being pH-responsive. The term “biomaterials” is utilized throughout the manuscript for the lipids and copolymers due to their biomimetic characteristics, as well as their usage as components of nanosized drug delivery systems, their ability to self-assemble spontaneously in aqueous environment, and their biocompatibility based on an in vitro cytotoxicity assay [27]. Special attention is given to the design elements that might have an impact on their behavior, such as the lipid composition, the % comonomer ratio, and the lipid to polymer ratio. In our previous publication, we examined the cooperativity between the biomaterials and how the aforementioned design parameters influence the thermodynamic (in a solid state), physicochemical, and toxicological features of DSPC:DOPC:P(OEGMA-co-DIPAEMA) hybrid nanostructures. Briefly, the variant systems exhibited different pH responsiveness and biocompatibility depending on the lipid(s) type (DSPC or DSPC:DOPC mixture). A similar but inverse effect was observed regarding the cytotoxicity for hybrid nanoparticles of P(OEGMA-co-DIPAEMA) with DIPAEMA as the predominant comonomer. Moreover, in both cases, the different lipid to polymer ratio led to different physicochemical and stimuli-responsive properties [27]. According to these results, specific systems were selected for further evaluation and drug delivery application. To the best of the authors’ knowledge, this is the first time that P(OEGMA-co-DIPAEMA)/lipid hybrid colloidal dispersions are being investigated from a membrane mechanics, biophysical, and morphological perspective. Keeping in mind the added value of hybrid lipid/polymer nanoparticles for the delivery of MTX, we used it as a model API in order to prove the capacity of the prepared systems to load and deliver it into cell lines. Therefore, in vitro evaluation was also conducted, providing encouraging results for lipid/random copolymer therapeutics. The scientific significance of the present study involves the investigation of complex interactions between different in-nature biomaterials, namely phospholipids and copolymers with random topology and stimuli-responsive properties from different perspectives, with the intention to be used as prototypical systems for the rational design of effective and smart drug delivery systems for spatiotemporal release.

## 2. Materials and Methods

### 2.1. Materials

**Figure 1:**
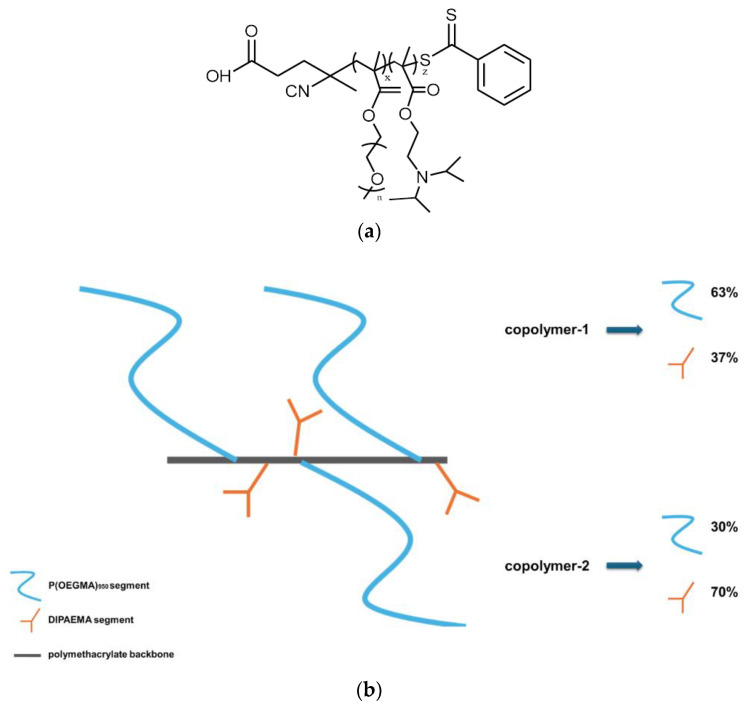
(**a**) The chemical structure of the random copolymer P(OEGMA950-co-DIPAEMA) synthesized by RAFT polymerization; (**b**) Graphic illustration of P(OEGMA-co-DIPAEMA)-1 or copolymer 1 and P(OEGMA-co-DIPAEMA)-2 or copolymer 2, respectively, with a different % comonomer ratio.

All reagents, being of analytical grade, as well as the fluorescent probe 6-dodecanoyl-N,N-dimethyl-2-naphthylamine (Laurdan) and methotrexate (MTX), in the form of a yellow powder, were obtained from Sigma–Aldrich Chemical Co, St. Louis, MO, USA. Fetal bovine serum (FBS) and phosphate buffer saline (PBS) tablets were purchased from Sigma–Aldrich Chemical Co, St. Louis, MO, USA, and Fisher BioReagents, Global Chemicals, Thermo Fisher Scientific, Pittsburgh, PA, USA, respectively. DSPC and DOPC phosphatidylcholines were purchased from Lipoid GmbH, Ludwigshafen, Germany. RAFT polymerization was utilized for the random copolymer synthesis. For this chemical reaction, 4-cyano-4-(phenylcarbonothioylthio) pentanoic acid (CPAD) was used as the chain transfer agent (CTA), azobis(isobutyronitrile) (AIBN) as the radical initiator, and 1,4-dioxane as the solvent. All chemicals and materials were added in appropriate amounts to a one-necked, round-bottom flask with a magnetic stir bar. The flask was fitted with a rubber septum, and the mixture was placed to degas for 20 min by nitrogen flow. Afterwards, it was left in a controlled temperature oil bath at 70 °C for 24 h, and then the flask was placed at −20 °C for 15 min. Subsequently, the reaction mixture was exposed to air, and a large amount of n-hexane was added so that the unreacted monomers would be removed and the polymer product of the reaction would precipitate. Finally, the copolymer was left to dry for 48 h in a vacuum oven. We expect the copolymers to be of a statistical/random architecture due to the methacrylate nature of the comonomers and their copolymerization in a solution mixture. We have not performed detailed copolymerization studies to determine the reactivity ratios of the particular pair of monomers, and we cannot define the exact comonomer sequences in the polymer chain. More information about the copolymers and their chemical characterization can be found in Table S1 and in our previous publication [27], whereas their chemical structure and graphic illustration are presented in Figure 1.

### 2.2. Methods

#### 2.2.1. Formulation of Hybrid Systems

The biomaterials utilized were mixed in selected combinations according to our previous publication results, and hybrid systems were formed by the thin film hydration protocol followed by probe sonication for size reduction [27]. The thin film hydration method took place above the phase transition of the mixture (65 °C) so that the biomaterials would be in liquid form during hydration. Probe sonication was conducted at ambient temperature without the use of any kind of water bath. There were two sonication cycles, having a duration of 5 min each with a resting period (5 min) in between. In all cases, the colloidal concentration of the hybrid structures was equal to C = 5 mg/mL. The prepared hybrid systems were: DSPC:P(OEGMA-co-DIPAEMA)-1 in three different lipid to polymer weight ratios (9:1, 7:3, 5:5), DSPC:P(OEGMA-co-DIPAEMA)-2 in three different lipid to polymer weight ratios (9:1, 7:3, 5:5), DSPC:DOPC:P(OEGMA-co-DIPAEMA)-1 at a 9:1 lipid to polymer weight ratio, and DSPC:DOPC:P(OEGMA-co-DIPAEMA)-2 at a 9:1 lipid to polymer weight ratio. In the cases where the lipid part was a mixture of DSPC/DOPC lipids, the weight ratio of the lipid mixture was equal to 9:1.

#### 2.2.2. Dynamic Light Scattering (DLS)

The physicochemical properties of the hybrid systems the day of their preparation were examined by the dynamic light scattering technique and were monitored for a period of 28 days by evaluating the scattered light intensity (I), the hydrodynamic radius (R_h_), and the size polydispersity index (PDI). Briefly, 50 μL of concentrated stock solution and 2 mL of aqueous medium (water for injection) were added in a cuvette. The measurements were conducted on an ALV/CGS-3 Compact Goniometer system (ALV GmbH, Langen, Germany) at a fixed scattering angle of 90° and ambient temperature, while obtained correlation functions were processed by the CONTIN algorithm. Each experiment was performed for three independent samples. The in vivo stability study was carried out at room and body temperature, applying an equilibration period of 5 min. The dispersion medium used for this purpose was a mixture of FBS:PBS at a 1:9 volume ratio (biorelevant medium). More information on the equipment utilized can be found in the literature [27].

#### 2.2.3. Fluorescence Spectroscopy (FS)

The membrane properties of the hybrid systems were investigated by FS utilizing the Laurdan probe [28,29,30,31,32,33,34]. Laurdan can be incorporated into the hydrophobic regions of the lipid/polymer structures, giving information about the microfluidity [35] via the general polarization value (GP), which is a semi-quantitative measure. The GP value was calculated by the following equation:

(1) $$\mathrm{GP}=\frac{I_{440}-I_{490}}{I_{440}+I_{490}}$$

I_440_ and I_490_ are the emission spectrum intensities at the blue and red edges, respectively.

The experimental protocol included the preparation of an ethanol stock solution of Laurdan probe at a final concentration of 0.5 mM. Afterwards, 1 mL of the lipid/polymer solution was mixed with 5 µL of the probe and inserted into the sample cell after a 24 h rest period (4 °C). The measurements were performed at ambient temperature and at 37 °C with a 5 min incubation period utilizing a double-grating excitation and single-grating emission spectrofluorometer (Fluorolog-3, model FL3-21, Jobin Yvon-Spex, Horiba Ltd., Kyoto, Japan) using a quartz cell and detected at a 90° angle. The emission spectra were recorded in the range λ_em_ = 380–600 nm, utilizing an excitation wavelength of λ_ex_ = 340 nm.

#### 2.2.4. Cryogenic Transmission Electron Microscopy (cryo-TEM)

Cryogenic transmission electron microscopy (cryo-TEM) images were collected using a Tecnai F20 X TWIN microscope (FEI Company, Hillsboro, OR, USA) equipped with a field emission gun operating at an acceleration voltage of 200 kV. Images were captured on the Gatan Rio 16 CMOS 4k camera (Gatan Inc., Pleasanton, CA, USA). Processing was conducted by Gatan Microscopy Suite (GMS) version 3.31.2360.0 software (Gatan Inc., Pleasanton, CA, USA). The resolution of the instrumentation allows for an accuracy of several angstroms (based on manufacturer’s data). Specimens were prepared by vitrification of the aqueous solutions on grids with holey carbon film (Quantifoil R 2/2; Quantifoil Micro Tools GmbH, Großlöbichau, Germany). Prior to use, a Femto plasma cleaner (Diener Electronic, Ebhausen, Germany) was used so the grids were activated in oxygen plasma for a period of 15 s. For cryo samples preparation, a droplet (3 μL) of the suspension was applied to the grid, blotted with filter paper and followed by immediate freezing in liquid ethane using a fully automated blotting device Vitrobot Mark IV (Thermo Fisher Scientific, Waltham, MA, USA). After preparation, the vitrified specimens were kept under liquid nitrogen until they were inserted into a cryo-TEM-holder Gatan 626 (Gatan Inc., Pleasanton, USA) and analyzed in the TEM at −178 °C.

#### 2.2.5. Thermal Evaluation of Hybrid Systems Using mDSC and HR-US

Microcalorimetry (mDSC) and high-resolution ultrasound spectroscopy (HR-US) techniques were used to determine the thermodynamic and acoustic features of the hybrid systems in the colloidal state.

A microDSC III (Setaram, Lyon, France) was used for a calorimetric analysis of the hybrid structures, while the thermodynamic parameters, temperature (T_m_, °C) and enthalpy (ΔH, J/g of solution), were measured by the software of the instrument (Setsoft 2000, Setaram, Lyon, France) based on the tangent method. The experimental data were also normalized to the overall sample mass so that the thermodynamic parameters were independent of the mass and were comparable with each other. All experiments were conducted in triplicates. The exact protocol can be found elsewhere [36,37]. Regarding HR-US, the ultrasound parameters were displayed as a function of temperature using a HR-US 102 high-resolution spectrometer (Ultrasonic Scientific, Dublin, Ireland). Ultrasonic cells were filled with 2 mL of lipid/polymer solution and the reference was HPLC-grade water. The thermal program of choice was the same as in the mDSC analyses [38]. Selected systems were investigated in acidic environment as well via both techniques. The pH of these hybrid lipid dispersions was adjusted to 4.5 using lactic acid before the analysis.

#### 2.2.6. Preparation and Characterization of Lipid/Copolymer Systems Incorporating MTX

Selected systems, namely DSPC:P(OEGMA-co-DIPAEMA)-2 at a 9:1 lipid to polymer weight ratio and DSPC:P(OEGMA-co-DIPAEMA)-2 at a 5:5 lipid to polymer weight ratio were chosen for model API loading. According to our previous and current results, these systems are distinguished from the rest of the formulations due to an adequate size for IV administration and better physicochemical stability. Additionally, they exhibited differences in their performance that could lead to valuable information about the hybrids’ properties. In particular, they showed differences regarding the physicochemical features; the calorimetric characteristics, especially in an acidic environment; the gamut of morphologies; and the cytotoxicity [27]. The preferred MTX concentration was equal to 0.2 mg/mL. MTX was dissolved in chloroform and added in the preparation that was processed according to the protocol described in Section 2.2.1. The MTX-containing hybrid systems were characterized for their physicochemical properties on the day of their preparation, and their stability was observed during 21 days via DLS. The protocol for the DLS technique is mentioned in Section 2.2.2. The MTX samples were also filtered before the measurement using hydrophilic Millipore^®^ syringe filters with a pore size of 0.22 μm.

#### 2.2.7. MTX Loading and Release Studies

The MTX entrapment efficiency was determined by UV-Vis spectroscopy (Perkin-Elmer Lambda 19 UV-Vis spectrophotometer, Waltham, MA, USA) that measured the absorbance at a wavelength of 303 nm [39,40,41,42]. Briefly, a 50 μL sample solution was diluted in DMSO into a quartz cuvette at a final volume of 3 mL. The EE% was determined according to the following equation:

(2) $$\mathrm{EE}\%=\frac{\;\mathrm{MTX}\;\mathrm{loaded}\;\mathrm{concentration}}{\mathrm{initial}\;\mathrm{MTX}\;\mathrm{concentration}\;\mathrm{used}}*100$$

The MTX-loaded concentration is the one detected in the final hybrid colloidal dispersion, which was calculated based on an MTX calibration curve. The experiment was conducted in triplicate.

#### 2.2.8. In Vitro Efficacy

For the experimental needs of this study, HeLa cervical carcinoma and HEK293 normal human embryonic kidney cells from ATCC (HeLa CRM-CCL-2 TM, HEK293: HTB-22TM) (LGC Standards GmbH, ATCC, Wesel, Germany) were cultivated in Dulbecco’s modified Eagle’s high-glucose medium (DMEM) (Gibco BRL, Life Technologies, ThermoScientific, Paisley, UK), supplemented with 10% FBS and antibiotics (1% penicillin/streptomycin) (Gibco BRL, Life Technologies, Thermo Scientific, Paisley, UK), at 37 °C and 5% CO_2_ [43,44].

The cytotoxicity of MTX-DSPC:2 9:1 and MTX-DSPC:2 5:5 was investigated, employing the MTT (3-(4,5-dimethylthiazol-2-yl)-2,5-diphenyl-tetrazolium bromide) colorimetric assay (Thiazolyl Blue Tetrazolium Bromide M5655, Sigma-Aldrich, Darmstadt, Germany) and utilizing a spectrophotometer for the quantification of cell viability by measuring the optical density of each sample. More specifically, the cells were seeded at approximately 9000–10,000 cells/well in 96-well plates and treated with increasing concentrations of MTX-DSPC:2 9:1 and MTX-DSPC:2 5:5, ranging from 0 to 50 μg/mL. On the day of the MTT assay, the culture medium in each well was replaced with fresh medium. A 10 μL amount of MTT solution (concentration 5 mg/mL in phosphate-buffered saline (PBS) (Gibco BRL, Life Technologies, ThermoScientific, Paisley, UK) was added to each well. Samples were incubated at 37 °C for 2 h. Afterwards, the supernatant was removed, and 100 μL of dimethyl sulfoxide (DMSO) was gradually supplemented in each well, and the plates were incubated on a shaker for 30 min at room temperature (RT). The optical density was measured at 570 nm and 650 nm for background normalization. The percentage of cell viability was calculated and compared with those of the untreated control samples [43,44,45]. Statistical analysis was applied through the Kruskal–Wallis non-parametric test, and *p* < 0.05 was considered statistically significant.

## 3. Results and Discussion

### 3.1. Physicochemical Evaluation of Lipid/Copolymer Colloidal Dispersions

**Figure 2:**
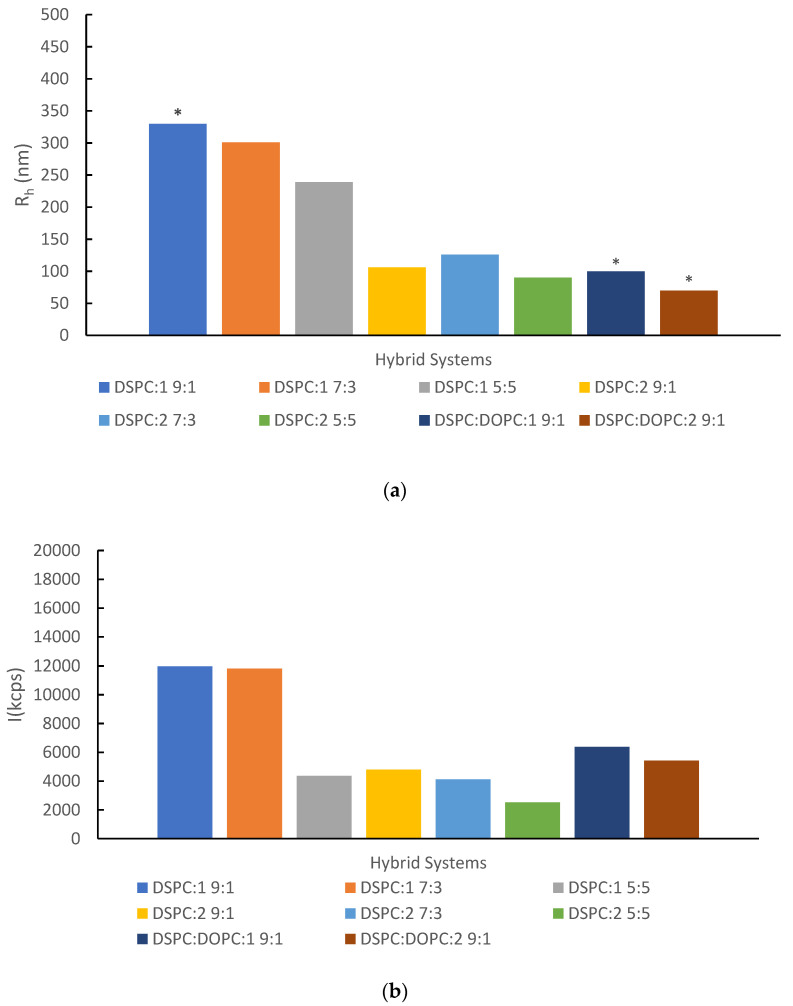
Charts derived from DLS measurements at 25 °C: (**a**) The hydrodynamic radius (R_h_, nm); (**b**) the scattered intensity (I, kilocounts per second or kcps) of hybrid colloidal dispersions the day of their preparation, utilizing water for injection as the dispersion medium. The standard deviation (SD) is less than 10% in both diagrams. * Hybrid systems with more than one population; the predominant (higher intensity) one is presented in the graph.

**Figure 3:**
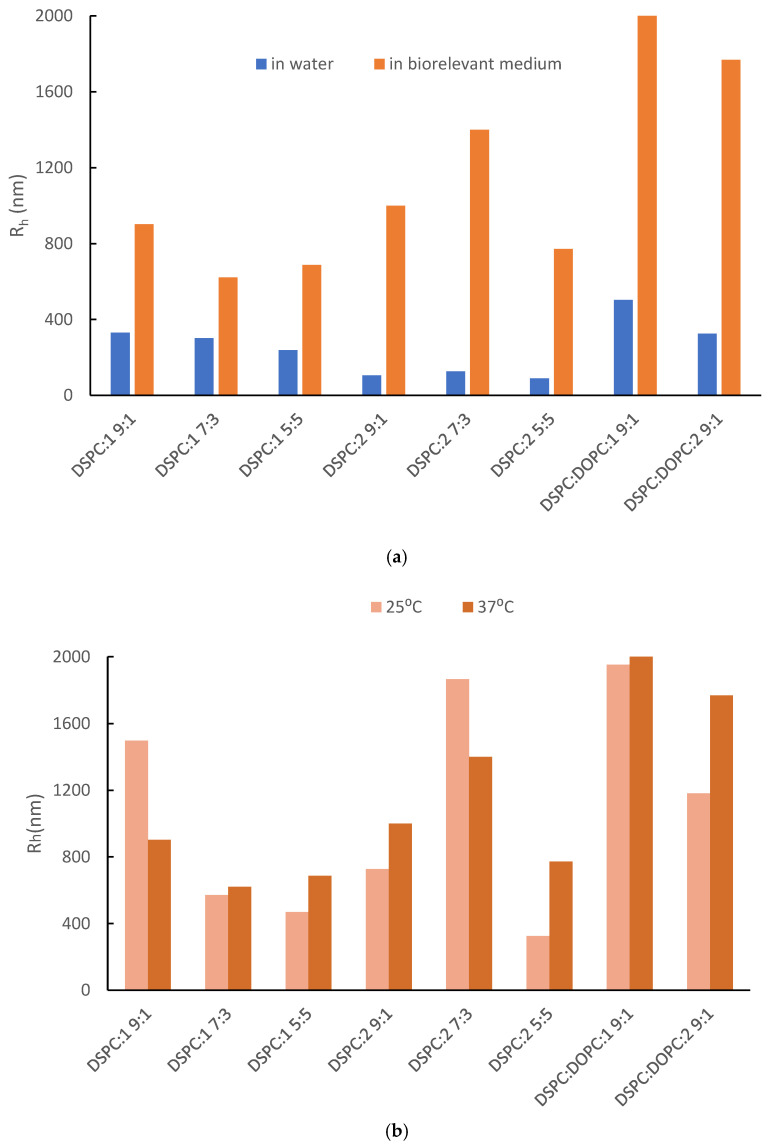
The hydrodynamic radius (R_h_, nm) of the hybrid colloidal dispersions in: (**a**) different dispersion media at body temperature (37 °C); (**b**) FBS:PBS biorelevant medium at different temperatures. The standard deviation (SD) is less than 10% in both diagrams. The DSPC:DOPC:1 9:1 hybrid system at 37 °C in both diagrams refers to a very high R_h_ compared with the rest of the systems exceeding the scale of the graph.

**Figure 4:**
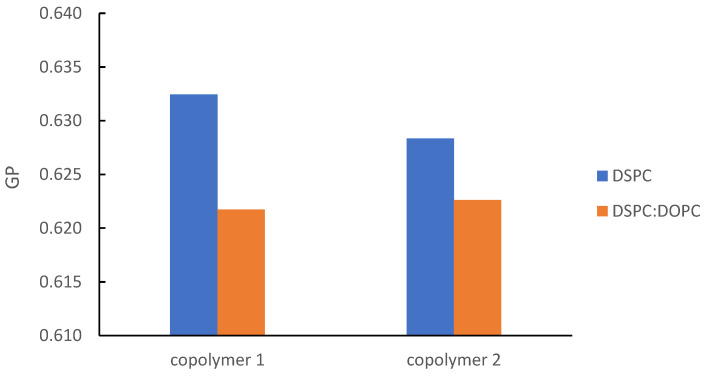
GP parameter vs. lipid composition of P(OEGMA_950_-co-DIPAEMA) hybrid systems at a steady lipid to polymer weight ratio (9:1).

After preparation, the hybrid lipid/copolymer systems were investigated for their physicochemical characteristics by DLS and FS. Stability studies in biorelevant medium (FBS:PBS at room and body temperature) or over time (a period of 28 days) were also performed to evaluate their physicochemical stability in simulated physiological conditions or during storage, respectively. The results are illustrated in Figure 2 and Figure 3. Fluorescence spectroscopy utilizing a Laurdan probe enabled the microfluidity assessment, and the relevant data are shown in Figure 4 and Table S2. Moreover, due to their thermosensitivity, the systems were examined at two different temperatures (25 and 37 °C) [27]. Further information about the physicochemical characteristics can be found in the Supplementary Materials as well (Tables S2–S4).

**Figure 5:**
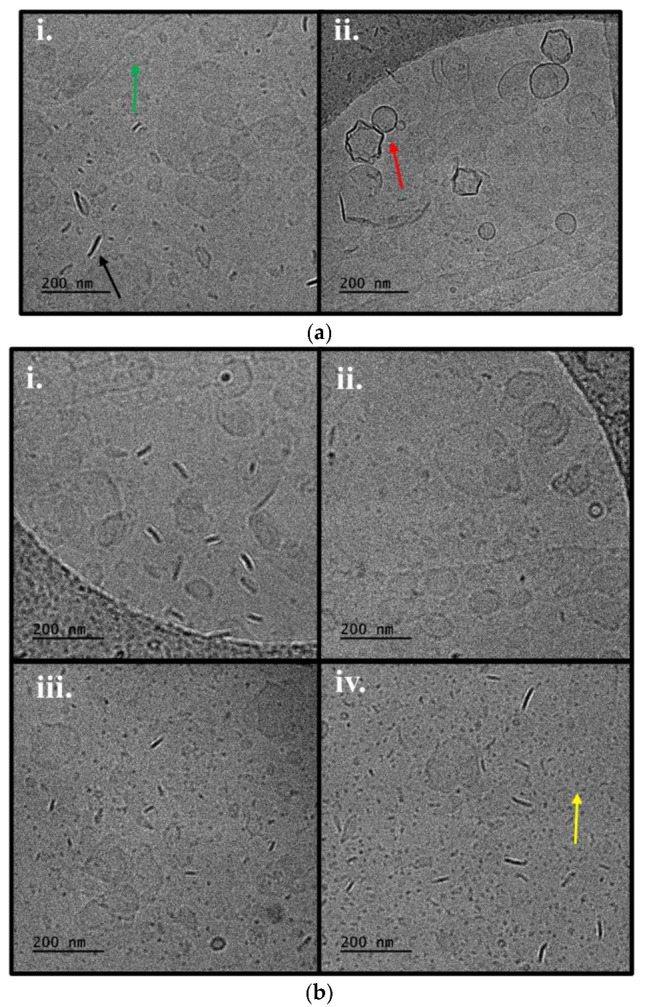
Cryo-TEM images of (**a**) P(OEGMA-co-DIPAEMA)-1. (**b**) P(OEGMA-co-DIPAEMA)-2 hybrid systems with different lipid compositions: (**i**) DSPC; (**ii**) DSPC:DOPC (9:1 weight ratio) and constant lipid to polymer ratio (9:1) or a constant lipid composition (DSPC) with different lipid to copolymer weight ratios: (**iii**) 7:3 and (**iv**) 5:5. The arrows represent the following: green color: spherical or irregularly shaped particles with distinct membrane; red color: “patchy” spherical- or pentagon-shaped vesicles; black color: rods; yellow color: small spherical particles.

The DSPC:P(OEGMA-co-DIPAEMA)-1 9:1 hybrid system presented two size populations in an equal intensity ratio; in particular, R_h_ is equal to 71 and 330 nm. The polydispersity index (0.48) reflects the heterogeneity of the system as well. Nevertheless, the larger population predominates with the increase in the % weight content of copolymer into the hybrid system, while the scattered intensity—which is proportional to the mass of the colloidal systems—is significantly decreased for DSPC:P(OEGMA-co-DIPAEMA)-1 5:5, but not in a similar manner to the size reduction [46,47]. A less compact structure of the assemblies may be responsible for this phenomenon due to the increase in the random copolymers amount and by extension due to more entry and exit points in the structure [38]. The utilization of a larger amount of DIPAEMA resulted in a different co-assembly of the hybrid structures, favoring the formation of smaller nanoplatforms regarding the hydrodynamic radius and intensity, as well as a narrower size distribution. Morevoer, the DSPC:P(OEGMA-co-DIPAEMA)-2 5:5 system seems more compact with a homogenous population. The addition of DOPC in both cases was accompanied by heterogeneity in size, as discerned by the presence of two populations. An interesting observation refers to the size populations of the DSPC:DOPC:P(OEGMA-co-DIPAEMA)-2 system, which are almost the same as the DSPC:P(OEGMA-co-DIPAEMA)-1 ones. Even though the scattered light intensity value is much lower, the similarity in size corresponds to the morphologies observed by cryo-TEM (Figure 5). In our opinion, the steric effects due to the increase in DIPAEMA segments likely counterbalance the DOPC fluidization effect having a great impact on the hydrophobic to hydrophilic balance, as well as the interactions between the biomaterials and the interfacial tension of the surface.

**Figure 6:**
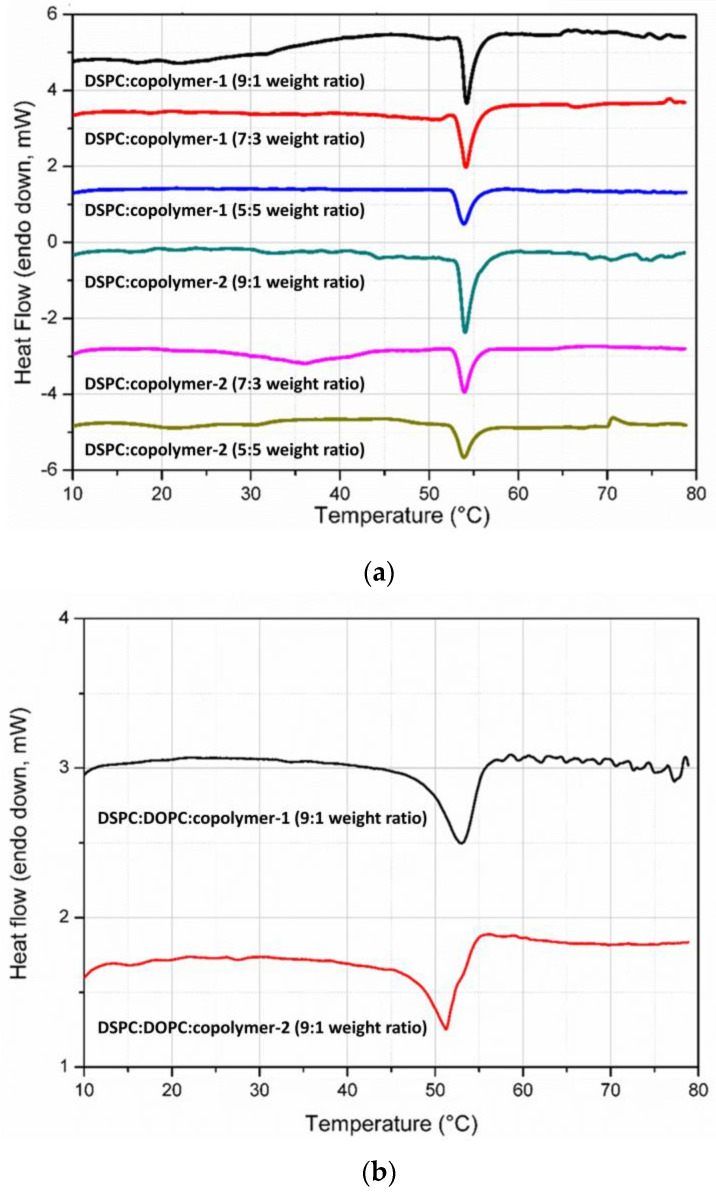
mDSC traces of (**a**) DSPC; (**b**) DSPC: DOPC (9:1 weight ratio) hybrid systems integrating P(OEGMA-co-DIPAEMA)-1 or -2 at different lipid to polymer weight ratios into aqueous medium.

As can be seen in Table S3 and Figure 3a, the incubation of the hybrid systems in simulated physiological conditions in FBS:PBS increased R_h_, PDI, and scattered intensity (I). This could be attributed to the protein corona formation, a new morphology due to the adsorption of serum proteins onto the nanoparticles [48,49,50]. This supramolecular morphology in most cases has a negative impact on the nanoparticle functionality and fate in vivo [51,52,53,54]. Typically, the presence of OEGMA chains on the exterior region of nanoparticles could give a solution to this problem due to diminished interactions with the opsonins in vivo [55,56,57,58]. However, this is not the case for the prepared hybrid systems, probably due to a non-ideal OEGMA chain configuration. Additionally, the in vivo behavior of nanoparticles can be quite challenging to determine as it depends on multiple parameters (e.g., size, shape, composition etc.) [54,59,60,61,62]. Considering the small differences between the hydrodynamic radii in Figure 2a and Figure 3a (blue bars), this could be attributed to the systems’ thermosensitivity. However, thermoresponsiveness in aqueous medium was mainly examined in our previous publication [27]. In this work, we focused on its impact on biorelevant conditions and protein corona formation. For this purpose, the systems were examined for their behavior in the serum environment at two different temperatures due to potential thermoresponsiveness; the protein corona is formed at both temperatures. The dynamics of the protein binding and the physicochemical properties of the system differentiate in ambient and body temperature with no specific patterns though (Figure 3b, Table S3). It is worth mentioning that the size of DSPC:DOPC:1 9:1 seems to strongly depend on temperature (Figure 3b). According to the thermal analysis (Section 3.3), the addition of DOPC in the DSPC:P(OEGMA-co-DIPAEMA) hybrid systems led to different biophysical behavior (Figure 6 and Table S5). The same observation also applies for DSPC:DOPC:1 compared with DSPC:DOPC:2 regarding T_m_ and enthalpy (Table S5). Moreover, the interactions of the serum proteins with the present nanoplatforms are a dynamic and multifactorial phenomenon that is affected by OEGMA chains conformation as well [60]. Keeping in mind the thermoresponsiveness of the copolymers, we could assume that the mixing of different biomaterials is resulting in peculiar molecular interactions and thus a different configuration of the components into the mixed structure and co-assembly. In this manner, the system exhibits unique characteristics, leading to a respective distinct response to temperature alterations and supramolecular morphology. In our opinion, other influential factors could be the random molecular topology of copolymers and the hydrophilic to hydrophobic balance, leading to the aforementioned peculiar interactions between the biomaterials with each other and with serum proteins as well.

According to the stability study, the colloidal systems did not preserve their properties for 28 days (Table S4). However, there are some interesting observations. First, DSPC:P(OEGMA-co-DIPAEMA)-2 5:5 was stable in storage conditions for at least 3 weeks (Figure S1). Although DLS measurements of DSPC:P(OEGMA-co-DIPAEMA)-1 9:1 did not correspond to a thermodynamically stable system, the small-sized population maintained its dimensions for at least two weeks with no deviations. Another observation that is worth mentioning refers to DSPC:P(OEGMA-co-DIPAEMA)-1 and -2 systems at a lipid to polymer ratio of 7:3. Their size changed from the day of their preparation but afterwards remained almost stable for at least two weeks. This could be accredited to conformational adaptation, which was favored thermodynamically according to the extended DLVO theory [63].

Both studies indicated the physicochemical instability of most of the hybrid systems in serum and/or storage conditions. Comparing our previous and current results, it can be concluded that there are size variations that could be correlated to the scale-up procedure [27]. In this manner, the instability issues could be due to the increased particle size and should be further examined.

Regarding the microfluidity of the systems, utilization of Laurdan confirmed the fluidization effect of DOPC integration into the hybrid systems (Figure 4) at 25 °C. The lipid composition is an important factor regarding the membrane packing, while neat DOPC liposomes exhibit negative GP values [31,34].

The increase in DIPAEMA content in the DSPC hybrid systems at a constant lipid to polymer ratio demonstrated a lower GP (Table S2). This is reasonable due to the DIPAEMA component, which in an aqueous environment is partially protonated and not completely charged [64,65]. The DSC results verify this assumption in accordance with the enthalpic reduction. A similar pattern took place via increasing the % weight of P(OEGMA-co-DIPAEMA)-1 in the colloidal systems. The only exception refers to system with the lipid to polymer ratio of 5:5. In this case, a more rigid membrane was formed for DSPC:P(OEGMA-co-DIPAEMA)-2. A possible explanation is the steric hindrance of the DIPAEMA component, which is present in a greater proportion. According to DLS measurements, this could also be attributed to the increased surface curvature due to the formation of smaller particles [34]. Certainly, the magnitude of effects caused by the random topology of copolymers is highlighted once more by raising unique characteristics to each system. Keeping in mind that different interactions lead to different membrane interfacial tension as discussed in the cryo-TEM section, the variability of GP constant is reasonable [66]. The change in temperature is another parameter that had an impact on the P(OEGMA-co-DIPAEMA) hybrid systems, mainly leading to more disordered packing and ascertaining the thermosensitivity of these hybrid systems (Table S2).

### 3.2. Morphological Characteristics of the Lipid/Copolymer Structures

Selected lipid/copolymer systems were probed via cryo-TEM to elucidate their morphological characteristics. Interestingly, morphological diversity is observed (Figure 5) and is summarized in Table 1 with more details on their dimensions.

The membrane thickness could provide additional information on the assembly of membrane components. According to the literature, a lipid bilayer thickness is usually equal to or less than 5 nm, although there are cases that it is equal to 6 nm. It might be dependent on the lipid composition, while in hybrid lipid/copolymer bilayers, the quantity of each component and the conformation of the copolymer into the bilayer play a crucial role on the membrane thickness [36,67,68,69,70,71,72]. Our results indicate the successful incorporation of random copolymers into the lipid bilayer and the hybrid nature of the resulting membrane having an 8–10 nm wall thickness (Table 1). In some cases, there are also vesicular structures of 6 nm thick membrane walls. We believe that these might correspond to hybrid morphologies as well, but with a different copolymer composition or configuration. Although the colloidal dispersions show respective membrane thickness, in fact, each hybrid system exhibits unique properties regarding the size and the morphology. This is reasonable due to the phenomena affecting the membrane tension, leading to a different co-assembly and by extent differently shaped structures, involving the interfacial curvature and entropy. The well-known influential parameters in lipid/block copolymer systems include the hybrid nature of the biomaterials and the critical packing parameter [67,68,69,70,73]. To the best of our knowledge, our research group is the first one studying the properties of hybrid nanocarriers composed of lipids and random copolymers for potential drug delivery. Keeping in mind the random distribution of hydrophilic/hydrophobic parts on these copolymers and the different comonomer ratios used, we are convinced that hybrids with unique characteristics are developed due to a different hydrophilic to hydrophobic balance and the random copolymer monomer sequence together with the graft macromolecular architecture, which are crucial factors for determining the morphology of the hybrid structures as well [27,74,75].

Despite the gamut of structures and the differences in size, there are common morphologies in all colloidal dispersions investigated: particularly, small spherical particles of 8–15 nm, spherical or irregularly shaped particles, and rods (Figure 5—yellow, green, and black arrow, respectively). In our opinion, the small spherical particles could be neat polymeric micelles due to the tendency of amphiphilic random copolymers to self-fold and build single- or multi- chain aggregates [76,77,78]. In addition, this assumption is in good agreement with the results of our previous publication, where populations of respective sizes were measured by light scattering techniques [27]. The spontaneous self-assembly of copolymers into micelles could lead to another structure when combined with lipids: the disk-like structure. This common morphology in hybrid lipid/polymer platforms resembles a liposome, but it is an open configuration and is correlated to the preparation procedure, and when it is observed from an edge-on orientation, it looks like a rod [79,80,81,82,83,84,85].

Regarding the peculiarity of its system, DSPC hybrids incorporating P(OEGMA-co-DIPAEMA)-2 instead of 1 show particles with pentagonal or hexagonal shape and spherical/irregularly shaped vesicles as well, while the size of the common morphologies is a bit smaller (Table 1). Considering the DOPC addition, spherical- or pentagon-shaped vesicles exist in DSPC:DOPC:copolymer-1 system that are “patchy”, as can be seen in Figure 5b(i) (red arrow). This could be attributed to nanodomain formation due to the fluid lipid DOPC phase and/or copolymer presence [67,86]. Additionally, DSPC:DOPC:P(OEGMA-co-DIPAEMA)-1 consists of structures analogous to DSPC:P(OEGMA-co-DIPAEMA)-2, whereas DSPC:DOPC:P(OEGMA-co-DIPAEMA)-2 dispersion lacks faceted particles. This extraordinary observation might imply that the increase in hydrophobicity up to a specific threshold favors the polygonal-shaped particles formation. These kinds of objects (faceted) are well known in hybrid systems composed of lipids and polymers. Moreover, they could be correlated with microdomains or the existence of rafts. This could be attributed to the inhomogeneous distribution of the random copolymer into the membrane [75,79,85]. Domains are deemed very important, not only for the functionality of biological membranes but also for drug encapsulation and release as well [85,87]. Moreover, the rod- and polygonal-shaped structures can be internalized into the cells easier and more effectively due to the increased number of interactions with target cell receptors compared with spherical particles of the same size [88,89,90]. Another interesting observation involves the polygonal-shaped particles. Namely, in our previous publication, we examined the toxicity of hybrid systems with respective compositions. All systems that exhibited a good cytotoxicity profile present faceted particles in our current study. The toxicity of nanovectors is a multifactorial concept though, with size being one of the main influential factors [88,89,90]. In this manner, we cannot come to specific conclusions given that the particle size of our systems is mainly not repeatable compared with our previous measurements due to the scale-up, as mentioned in Section 3.1 as well [27].

Comparing size measurements extracted from DLS and cryo-TEM, there are similarities regarding spherical/irregularly shaped particles. However, there are also deviations, and each technique can provide important information due to the different experimental procedures followed [82,85,91]. The complexity of the nanoparticles dictates the utilization of multiple and sensitive enough techniques for their better characterization and understanding. To the best of our knowledge, this is the first time that the morphological features of hybrid structures comprised DSPC, DOPC, and P(OEGMA-co-DIPAEMA) random copolymers have been studied.

### 3.3. The Thermal Behavior of Lipid/Copolymer Dispersions

The hybrid colloidal dispersions were examined for their thermal properties by two different techniques, namely mDSC and HR-US. Both methods are well established for the biophysical analysis of nanoparticulate systems in a temperature-dependent manner via thermodynamic or ultrasound parameters, respectively [37,92,93]. Exploiting their sensitivity to detect thermal events such as the transition from gel to a liquid crystalline state of lipid bilayers, we investigated the features of hybrid systems in aqueous medium, and the results are summarized in Table S5 and Figure 6 and Figure S2.

All the samples show the characteristic endothermic peak of DSPC, which is associated with a temperature of 54 °C in line with the literature (Table S5, Figure 6). The sharp endothermic peak corresponds to the main thermal event, from the gel to liquid crystalline state of the lipids [94]. Apart from hybrid systems composed of DOPC, the temperature that the melting process is centered at is similar, with only slight differences despite the increase in copolymer amount in the colloidal dispersions. However, the sharpness of the main peak as well as the enthalpy values (J/g of solution) vary for each system. This is an indication for the existence of different interactions between the biomaterials, implying the successful incorporation of the copolymers into the lipid bilayer, whereas it seems that the copolymer does not interfere much in the internal conformation of the lipid chains; thus, the temperature of the main event is preserved [27]. The hybrid nature of the dispersions and the copolymer’s integration in the systems at a different level are further supported by the more or less decreased enthalpy in comparison with pure DSPC liposomes of the same concentration (5 mg/mL), as stated by Perinelli et al. (2022) [94].

Specifically, for DSPC:P(OEGMA-co-DIPAEMA)-1 at a lipid to polymer ratio 9:1, its main transition is located at 54.21 °C, and the enthalpy is about 0.192 J/g. By increasing copolymer proportion in the dispersion, a decreasing trend is demonstrated for both features while being more pronounced in the case of enthalpy (0.154 and 0.134 J/g for the lipid to polymer ratio of 7:3 and 5:5, respectively), signifying a reduction in the van der Waals interactions between the lipid chains [85]. Taking into consideration the chemical composition of this copolymer and the tertiary amine group, which is partially deprotonated, steric hindrance by the hydrophobic DIPAEMA in the interface could be the reason for modifications in the mechanical properties of the bilayer [95,96]. Referring to DSPC:P(OEGMA-co-DIPAEMA)-2 9:1, the increase in the content of the bulky DIPAEMA comonomer led to increased enthalpy (0.239 J/g) compared with copolymer-1, accompanied by a main peak centered at 54.03 °C—a fact that strengthens our previous assumption. There is also a similar pattern with copolymer-1 by increasing the % weight of copolymer-2. Even though the enthalpy of copolymer-2 at a lipid to polymer ratio 9:1 has a difference of 50 J/mol compared with copolymer-1, the presence of copolymers in increased proportion into the structures led to similar enthalpy for the respective lipid to polymer ratio, in particular, 0.157 and 0.132 J/g for the lipid to polymer ratios of 7:3 and 5:5, respectively. This interesting observation points out that the two copolymers occupy equivalent space into the structure regardless of the different hydrophobic to hydrophilic ratio. It is well known that amphiphilic statistical copolymers do not express strong hydrophobic or hydrophilic properties compared with copolymers with other topologies due to the randomly dispersed monomeric segments [97].

As far as DOPC addition is concerned, the main transition temperature is lower and is equal to 53.09 and 51.95 °C for DSPC:DOPC:P(OEGMA-co-DIPAEMA)-1 and DSPC:DOPC:P(OEGMA-co-DIPAEMA)-2, respectively. DOPC lipid has already been in the liquid crystalline state at the whole temperature range of the mDSC technique due to a very low T_m_, owing to a cis-double bond in its acyl chains [98,99,100]. In this manner, DOPC presence in the membrane of the structure provokes conformational freedom between the hydrocarbon chains; thus, more fluid bilayers are formed. The increased fluidity for these systems is also detected by the Laurdan probe during fluorescence spectroscopy experiments (Table S2, Figure 4). The enthalpic value for DSPC:DOPC:P(OEGMA-co-DIPAEMA)-1 is higher than that of the respective system without DOPC, accompanied by a more broad main peak (0.220 J/mol), whereas for P(OEGMA-co-DIPAEMA)-2, the exact opposite (0.175 J/mol) is observed. Given that enthalpy changes are associated with different inter- and intra-molecular interactions between the components [98], it is very likely—also considering the trends of the other thermodynamic parameters—that the increase in enthalpy in the former case is attributed to more intense interactions between the biomaterials in the hydrophobic interior. These interactions could be the result of nanodomains formation favored by the incorporation of the P(OEGMA-co-DIPAEMA)-1 in a lipid bilayer with a loose packing arrangement due to DOPC’s presence. Another reason for the observed enthalpy increase could be the existence of more hydrogen bonds between the copolymer and the phospholipid head groups [36].

Acoustic spectroscopy confirmed the abovementioned calorimetric characteristics due to comparable results obtained (Table S5). The changes in the velocity and energy of the propagation of the ultrasound waves in a temperature-dependent manner favored the effective detection of the main thermal event, as it is illustrated in Figure S2. Namely, by increasing the temperature, there is a pattern for both ultrasonic parameters (sound speed and attenuation), which deviates from its path only near the temperature of the main transition [94]. Notably, for the colloidal dispersions containing DOPC lipid, the deviations of the ultrasound features are not as steep as in the rest of the systems. Keeping in mind the ability of HR-US to detect structural and molecular events that could occur in the biomaterials, this could suggest different mechanical properties for these systems regarding density and compressibility, as well as heterogeneities in the bilayer [36,94].

**Figure 7:**
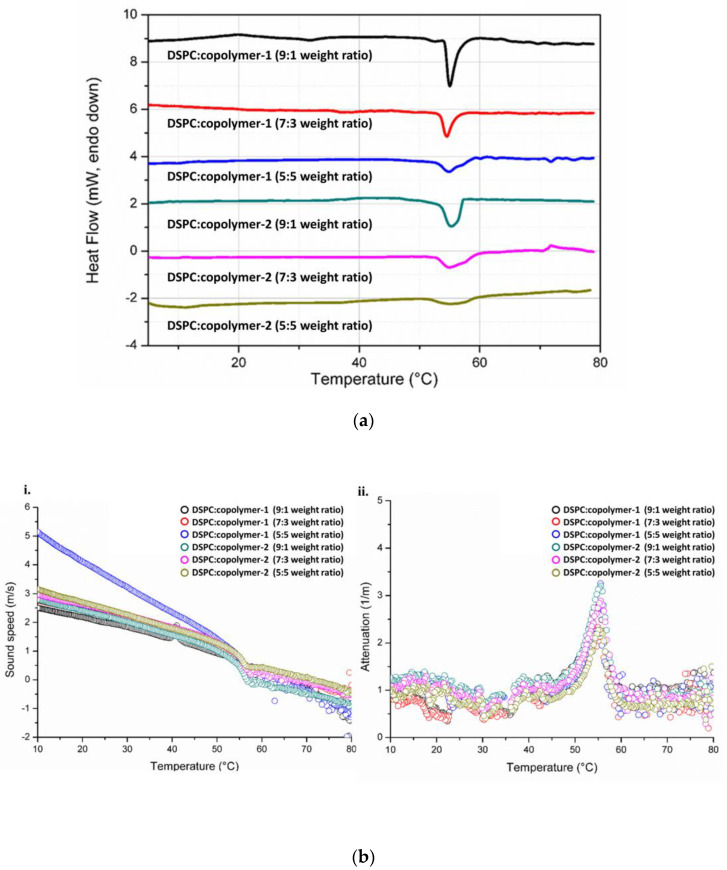
Thermodynamic evaluation of DSPC:P(OEGMA-co-DIPAEMA)-1 and -2 in an acidic environment (pH 4.5): (**a**) mDSC profiles; (**b**) (**i**) sound speed or (**ii**) attenuation vs. temperature from HR-US.

As can be observed in Table S5 and Figure 7, we also probed the biophysical response of selected systems in an acidic environment due to their pH sensitivity due to the presence of the tertiary amino groups of DIPAEMA [27].

All systems exhibit a pH-dependent thermodynamic behavior, which is more pronounced by increasing the copolymer amount and by incorporating copolymer-2 instead of -1 at a constant lipid to polymer ratio. This is obvious in the graphs obtained from both techniques, especially in Figure 7a. At pH 4.5, the peaks are broader, showing lower cooperativity between the biomaterials, variations in density and compressibility, as well as heterogeneities in the membrane [94,98]. The calorimetric parameters—especially the enthalpy—show differences as well, having a slightly higher value. This overall behavior could be the result of the protonated copolymer that is hydrophilic above its pKa (6.2), leading to different interactions between the biomaterials and possibly more intra- and inter-molecular hydrogen bonding [27,64,85,95,101]. Comparing our current to previous thermodynamic data, we could conclude that the main outcomes are confirmed, although there are differences in the experimental procedure and the state of the samples [27]. More importantly, the pH-dependent performance that was detected at the molecular level from mDSC and acoustic spectroscopy should be further investigated and correlated with the efficacy of respective hybrid drug delivery systems as it could be a valuable tool to passively target cancer cell lines (see Section 3.5).

### 3.4. Exploring Hybrid Lipid/Copolymer Platform Characteristics as Drug Delivery Systems

The necessity for innovative drug delivery platforms with sustained release capability and in-depth knowledge of their properties guided our attempt to develop a lipid/copolymer nanocarrier utilizing MTX as a model hydrophobic drug for potential anticancer therapy. For this purpose, and according to our previous and current results, we selected two systems that seem promising as drug nanovectors: namely, DSPC:P(OEGMA-co-DIPAEMA)-2 at two lipid to polymer ratios of 9:1 and 5:5. Our selection is based on their biocompatibility, which was recently confirmed by an MTS assay [27], and their differences in other features, such as morphological and biophysical, in order to unveil their characteristics and use them as prototypes in lipid/random copolymer nanoparticulate drug delivery systems. 

**Figure 8:**
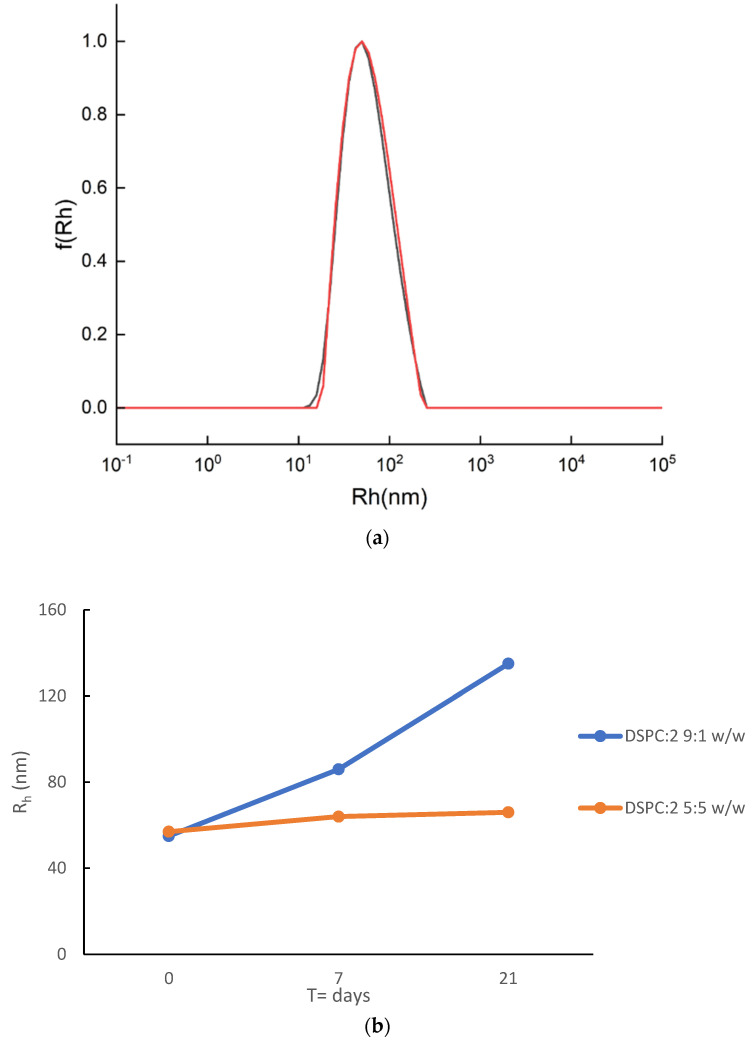
(**a**) Size distributions from the DLS of DSPC:P(OEGMA-co-DIPAEMA)-2 hybrid systems incorporating MTX at two different lipid to polymer ratios, 9:1 (black line) and 5:5 (red line), on the day of their preparation; (**b**) the systems’ stability assessment (R_h_ vs. time) under storage conditions (4 °C) for 21 days.

The prepared hybrid systems loaded with MTX were investigated for their physicochemical properties, stability in storage conditions (for 21 days), and %EE, and in vitro studies for their efficacy in various cell lines were also conducted. The initial results of the MTX-loaded hybrid systems can be found in Table S6 and Figure 8a, whilst the stability assessment is presented in Figure 8b and Figure S1. The in vitro data are discussed in the next section.

The UV-Vis measurements confirmed for the first time the successful incorporation of MTX in lipid/random copolymer hybrid carriers. Considering the %EE, both formulations showed a good encapsulation capability of more or equal to 84% (Table S6). However, it seems that the DSPC:P(OEGMA-co-DIPAEMA)-2 5:5 nanosystem had an increased capacity as a nanovector encapsulating MTX more efficiently (EE% = 100%). The DLS data demonstrated that MTX-loaded hybrid systems could be used as injectable formulations (e.g., intramuscular (IM) injection), having adequately homogenous populations (average PDI = 0.2), and their size did not exceed 120 nm (Table S6). Additionally, the small-sized nanoparticles could effectively accumulate in tumor areas through the EPR effect [102]. Even though the formulations differ from each other in their lipid to polymer weight ratio, their sizes were similar (Figure 8a). Their main difference concerns the mass of the colloidal system, being proportional to the measured intensity, their encapsulation efficacy, and their physicochemical stability (Table S6, Figure 8b). MTX-DSPC:P(OEGMA-co-DIPAEMA)-2 9:1 had a higher intensity compared with MTX-DSPC:P(OEGMA-co-DIPAEMA)-2 5:5, although the copolymer amount was lower. The scattered intensity was found to be inversely proportional to the lipid to polymer ratio as was also the case in the empty hybrid systems (Table S2) and our previous results as well [27]. The different intra- and inter-molecular interactions between the biomaterials involved, including the API, led to similar patterns with unloaded structures except for the size. Unloaded and loaded hybrid structures at a lipid to polymer ratio of 5:5 did not have an enormous difference in their features in comparison with the 9:1 ratio. In this case, the loaded systems show decreased physicochemical characteristics (at half) in all cases. On the contrary, based on our previous results for empty respective formulations, the incorporation of MTX in the hybrid systems led to comparable or slightly larger structures, and that is in line with the literature for liposomal and hybrid MTX nanocarriers [103,104]. In our opinion, it is the result of different co-assembly processes that led to empty and loaded systems having different properties; meanwhile, the scale-up is still an unsolved issue that should be further examined. Considering storage stability, the DSPC:P(OEGMA-co-DIPAEMA)-2 5:5 platform maintained its physicochemical features after API loading, exhibiting a stable size for at least 21 days (Figure 8 and Figure S1). On the other hand, the MTX loading and the different co-assembly of the biomaterials did not succeed in ameliorating the stability of DSPC:P(OEGMA-co-DIPAEMA)-2 9:1, which was found to be unstable within a week; a more than 50% increase in the size was observed (Figure 8b). The increased proportion of copolymers in MTX-DSPC:2 systems favored the physicochemical stability during time in accordance with the literature for lipid/polymer hybrids [11,13]. Considering the pH sensitivity, our former results exhibit a pH-dependent size fluctuation of the unloaded systems in the acidic environment (pH 1.2) [27], while both loaded systems ameliorated their size in pH 4.5 compared with the aqueous environment.

### 3.5. In Vitro Evaluation of MTX-Loaded Lipid/Copolymer Nanostructures

**Figure 9:**
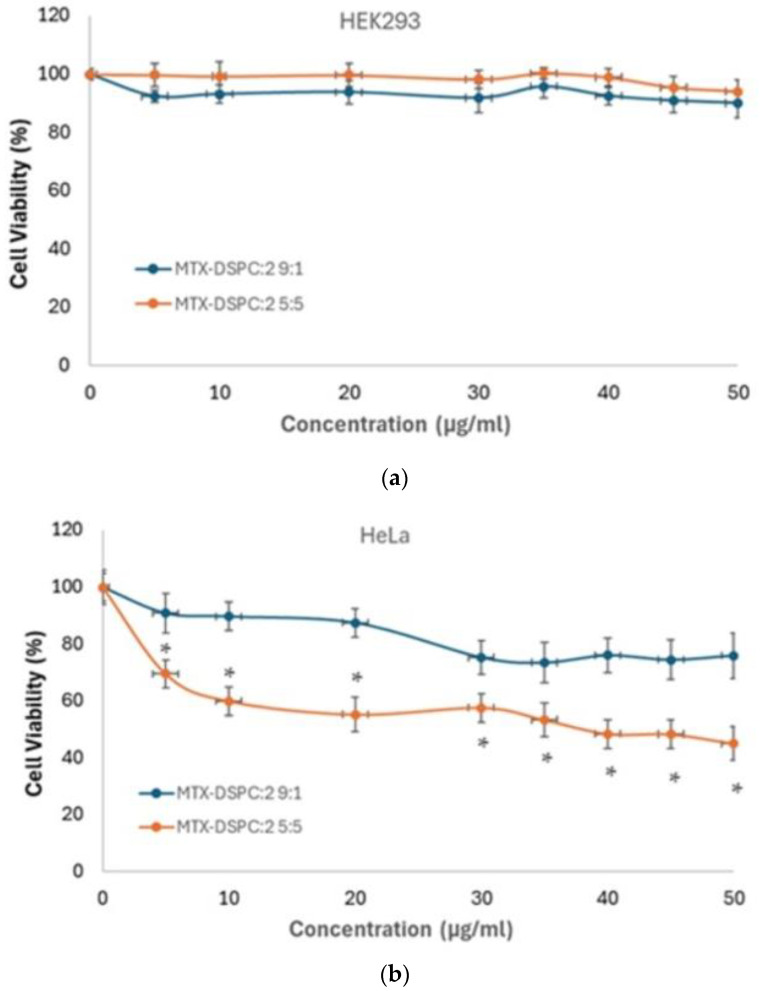
Cell viability vs. different concentrations of MTX-DSPC:2 9:1 (blue line) and MTX-DSPC:2 5:5 (orange line) on (**a**) HEK293 and (**b**) HeLa cells. The concentration levels refer to MTX concentration, and the obtained data represent the means ± standard deviation from three experiments conducted in triplicates. The asterisks (*) in (**b**) correspond to p values of less than 0.05 (*p* < 0.05) that are considered as statistically significant.

The MTX systems were examined for their in vitro effectiveness in cancer cell lines and their selectivity against normal ones. For this purpose, cell viability was estimated through an MTT colorimetric assay (Figure 9).

As it is shown in Figure 9a, there is not any significant effect on the viability of normal cells in the presence of each of the tested samples, MTX-DSPC:2 9:1 and MTX-DSPC:2 5:5, respectively. Considering that unloaded hybrid systems were biocompatible at small concentration levels according to our previous results [27], they managed to not only incorporate MTX but also probably screen the molecule in the interior of the structure, thus decreasing its toxicity. In other words, these results are of paramount importance because they revealed the biocompatibility of the carriers as well as their ability to encapsulate APIs by lowering their toxicity profile. The last observation is the formulation strategy of several anticancer APIs into nanocarriers that are in clinical use as nanomedicines [12]. Notably, MTX-DSPC:2 9:1 induced a slight decrease in cell viability in HeLa cancer cells (<20%) at concentrations of 30–50 μg/mL (Figure 9b). A statistically significant effect on HeLa was observed in the presence of MTX-DSPC:2 5:5, even at low concentrations. Particularly, MTX-DSPC:2 5:5 significantly decreased the cell population of HeLa by 30% at the concentration of 5 μg/mL by 40% at concentrations ranging between 10 and 35 μg/mL, while this effect became more intense for higher concentrations. Specifically, an amount of 40 μg/mL of MTX-DSPC:2 5:5 was proven to be cytotoxic for half of the HeLa cell population, and at 45 and 50 μg/mL, the cell viability was further decreased by ~55%. The results demonstrated the selectivity of MTX-DSPC:2 5:5 for cancer cells. More importantly, the MTX-DSPC:2 hybrid nanostructures at a lipid to polymer ratio 5:5 exhibited remarkable effectiveness at low concentrations of MTX and were superior to the respective system at a lipid to polymer ratio of 9:1 for cancer cell apoptosis. This outcome could be ascribed to the P(OEGMA-co-DIPAEMA) augmentation in the formulation leading to an increased pH sensitivity of the system [105]. The bimodal behavior in neutral and acidic conditions in combination with the small size of the system could be advantageous for site-specific release due to the tumor peculiar microenvironment and endosomal/lysosomal activity [106]. Interestingly, the different biophysical behavior of the two unloaded systems in the acidic environment (Figure 7) reflects on the loaded system potency in cancer cells. In our opinion, the gamut of mesophases and the low cooperativity between the biomaterials at an equal weight ratio at acidic conditions destabilize the structure and might correspond to the destruction of the hybrid system or the conversion to another morphology, thus leading to targeted API release into the lysosomes.

Moreover, the morphological differences of the systems is worth mentioning, particularly the faceted particles that were observed only in the case of the unloaded DSPC:2 9:1 system. Correlating this observation to our in vitro data, this extra morphology might have contributed to the insufficient efficacy of MTX-DSPC:2 9:1 on HeLa cell populations. However, considering the plethora of morphologies of the unloaded hybrid systems and the different co-assembly pattern by incorporating MTX, we cannot come to definite conclusions. Certainly, the examination of the MTX-loaded systems morphology in more detail could promote a better understanding of the systems as well as a better justification of this hypothesis.

## 4. Conclusions

DSPC:P(OEGMA-co-DIPAEMA) hybrid platforms of different % comonomer ratios, lipid to polymer ratios, and with the occasional presence of DOPC liquid lipid were examined for their morphological and thermodynamic features in a colloidal state for the first time. The fluidity of their membranes and their stability were also examined. Indeed, their morphology and membrane properties were influenced by the design parameters that were implemented and studied. The system parameters that stand out are the random architecture of the copolymers utilized and the hydrophobic to hydrophilic balance. The lipid composition is a considerable design factor as well, which reflects the membrane properties and the self-assembly of the hybrid systems during the preparation protocol. The lipid to polymer ratio does not seem to have a great impact on the morphological characteristics, although it is a crucial design factor for membrane mechanics and the thermotropic and physicochemical properties. Moreover, the different techniques confirmed the location of the copolymer into the bilayer for at least lamella morphologies, the existing pH- and thermo-responsive properties of the P(OEGMA-co-DIPAEMA) hybrid systems, and the DOPC fluidization effect as well. Previous and current results seem to be promising for spatiotemporal release, highlighting that hybrid DSPC:P(OEGMA-co-DIPAEMA) vectors could be useful in drug delivery to tumors where there are different inherent environmental characteristics.

Keeping in mind unmet patient needs, we develop step-by-step a lipid/random copolymer drug delivery system loaded with MTX for the first time to investigate the ability of the hybrid nanocarriers to incorporate a hydrophobic API as well as their stimuli-responsive ability. Based on our physicochemical and in vitro results, MTX-loaded DSPC:P(OEGMA-co-DIPAEMA) at a lipid to polymer weight ratio of 5:5 can be distinguished for its encapsulation capability, physicochemical stability, as well as in vitro selectivity and efficacy in HeLa cancer cells apoptosis, being a promising candidate for anticancer therapy.

In the present work, we studied DSPC:P(OEGMA-co-DIPAEMA) hybrid systems and highlighted the most influential parameters on their behavior, while we successfully loaded a model API into these systems, upgrading them into capable drug delivery vectors. The nature of the constructing biomaterials and their interactions with each other and the loaded API molecule as well set the way for a unique co-assembly and an overall exceptional behavior, including the structure, the physicochemical properties, and the thermodynamics of these hybrid systems. More importantly, the loading of an API could lead to different characteristics and intrinsic features; thus, they should be carefully investigated as well. Considering the lack of works in the literature regarding random copolymers with pH-responsive properties associating with phospholipids, our study aimed at the rational design and development of lipid/random copolymer hybrid systems with targeted release capability. In other words, the combination of the aforementioned materials exhibits dual significance: the preparation of compartmentalized drug delivery systems with pH-responsive properties, as well as polymer-grafted lipid particles with unique membrane properties, as calorimetry studies revealed. Last but not least, these systems can be used as a road map for the investigation of the biophysics of phospholipid membranes and the self-assembly of artificial protocells [107,108]. Despite the need for further investigation, especially regarding potency in vivo, we believe that the current study contributes to the elucidation of the complexity of hybrid lipid/copolymer drug delivery systems, adding another parameter into the equation: the one of the random copolymer topology.

## Figures and Tables

**Table 1:** Cryo-TEM measurement data of the different objects formed by DSPC or DSPC:DOPC (9:1 *w*/*w*) hybrid systems incorporating copolymer P(OEGMA_950_-co-DIPAEMA) (copolymer 1 or 2). The dimensional characteristics are presented in parentheses as diameter or rod length (nm), referring to the average size (based on observation of 50 objects).

Sample	*w*/*w*	Type of Objects ^1^				
		Small Spherical Particles ^2^	Particles with Spherical or Irregular ^3^ Shape	Particles with Nearly Pentagonal or Hexagonal Shape	Vesicles ^4^ with Spherical or Irregular Shape	Rods
DSPC:1	9:1	√	√ [20–500]	-	-	√ [20–100]
DSPC:DOPC:1	9:1	√	√ [20–300]	√	√ [30–300]	√ [20–70]
DSPC:2	9:1	√	√ [20–300]	√	√ [20–80] ^5^	√ [20–100]
DSPC:DOPC:2	9:1	√	√ [20–400]	-	√ [20–100]	√ [20–60]
DSPC:2	7:3	√	√ [20–300]	√	√ [20–70]	√ [20–60]
DSPC:2	5:5	√	√ [20–250]	-	√ [20–100] ^5^	√ [20–70]

^1^ For most of the examined objects, the wall thickness (or rods’ core diameter) is equal to 8–10 nm. ^2^ All small particle diameters are equal to 8–15 nm, and their wall thickness cannot be measured. ^3^ The measurement of the upper limit of sizes is difficult due to the irregular shape. ^4^ The vesicles wall thickness is equal to 6 nm. ^5^ A small number of these kinds of objects.

## Data Availability

Data are contained within the article and Supplementary Materials.

## Supplementary Materials

The following supporting information can be downloaded at: https://www.mdpi.com/article/10.3390/pharmaceutics16091204/s1, Table S1: The properties of the statistical (random) copolymers used in the present study; Table S2: Physicochemical properties of DSPC and DSPC:DOPC (9:1 weight ratio) hybrid systems incorporating copolymers P(OEGMA-co-DIPAEMA), utilizing water for injection as the dispersion medium and as measured on the day of their preparation; Table S3: DLS results of DSPC and DSPC:DOPC (9:1 weight ratio) hybrid systems incorporating stimuli-responsive copolymers P(OEGMA-co-DIPAEMA)-1 and -2 in FBS:PBS biorelevant dispersion medium at different temperatures; Table S4: Stability study of DSPC and DSPC:DOPC (9:1 weight ratio) hybrid systems incorporating stimuli-responsive copolymers P(OEGMA-co-DIPAEMA)-1 and -2; Table S5: Thermodynamic evaluation of lipid/copolymer colloidal dispersions in different environments (aqueous and pH 4.5) as measured by microcalorimetry (mDSC) and high-resolution ultrasound spectroscopy (HR-US); Table S6: Properties of hybrid DSPC:P(OEGMA950-co-DIPAEMA)-2 systems incorporating MTX on the day of their preparation at ambient temperature; Figure S1: Size distributions via the DLS of a. empty and b. an MTX-loaded DSPC:P(OEGMA-co-DIPAEMA)-2 hybrid system at a lipid to polymer weight ratio of 5:5 vs. time. Black line: t = 0 days, red line: t = 14 days, blue line: t = 21 days; Figure S2: Charts from HR-US: (a) sound speed; (b) attenuation vs. temperature for the i. DSPC and ii. DSPC:DOPC (9:1 weight ratio) hybrid colloidal dispersions into aqueous medium.
