# Supplementary material for: Unveiling the Performance of Co-Assembled Hybrid Nanocarriers: Moving towards the Formation of a Multifunctional Lipid/Random Copolymer Nanoplatform
Source: Pharmaceutics. 2024 Sep 13;16(9):1204. doi: 10.3390/pharmaceutics16091204 (PMC11434724; doi:10.3390/pharmaceutics16091204)
Supplement: Supplementary file 1 [file pharmaceutics-16-01204-s001.zip › pharmaceutics-3118203-supplementary.pdf]

Supplementary Materials

Article

Unveiling the performance of co-assembled hybrid nanocarriers: moving towards the formation of a multifunctional lipid/random copolymer nanoplatform

Efstathia Triantafyllopoulou, Diego Romano Perinelli, Aleksander Forys, Pavlos Pantelis, Vassilis G. Gorgoulis, Nefeli Lagopati, Barbara Trzebicka, Giulia Bonacucina, Georgia Valsami, Natassa Pippa* and Stergios Pispas*

Table S1. The properties of the statistical (random) copolymers used in the present study.

Copolymer	Abbreviation	M_w[1] ($x10^4$) (g/mol)	M_w/M_n[1]	wt%[2] OEGMA
P($OEGMA_{950}$-co-DIPAEMA)-1	copolymer 1	1.10	1.16	63
P($OEGMA_{950}$-co-DIPAEMA)-2	copolymer 2	1.24	1.13	30

[1] SEC in THF at 25°C
[2] ^{1}H-NMR in $CDCl_3$
OEGMA: Oligo (ethylene glycol) methyl ether methacrylate
DIPAEMA: 2-[diisopropylamino] ethyl methacrylate

Table S2. Physicochemical properties of DSPC and DSPC:DOPC (9:1 weight ratio) hybrid systems incorporating copolymers P(OEGMA-co-DIPAEMA) utilizing water for injection as the dispersion medium and measured the day of their preparation.

Sample	w/w	I (kcps)	R_h (nm) *	PDI	$GP_{25°C}$	$GP_{37°C}$
DSPC: copolymer 1	9:1	11968	a) 71 (45%), b) 330 (54%)	0.48	0.632	0.622
DSPC: copolymer 1	7:3	11806	301	0.48	0.629	0.619
DSPC: copolymer 1	5:5	4360	239	0.43	0.626	0.640
DSPC: copolymer 2	9:1	4810	106	0.43	0.628	0.626
DSPC: copolymer 2	7:3	4130	126	0.35	0.627	0.623
DSPC: copolymer 2	5:5	2519	90	0.24	0.632	0.619
DSPC:DOPC:copolymer 1	9:1	6380	a) 100 (52%), b) 503 (41%)	0.35	0.622	0.610
DSPC:DOPC:copolymer 2	9:1	5430	a) 70 (57%), b) 325 (42%)	0.47	0.623	0.616

*Percent in parenthesis represents the intensity weight of the main peak reported in the table for systems exhibiting more than one peaks.

Table S3. DLS results of DSPC and DSPC:DOPC (9:1 weight ratio) hybrid systems incorporating stimuli-responsive copolymers P(OEGMA-co-DIPAEMA)-1 and -2 in FBS:PBS biorelevant dispersion medium at different temperatures.

Sample	w/w	T (°C)	I (kHz)	R_h (nm) *	PDI	Number of peaks
DSPC: copolymer 1	9:1	25	14355	1498 (61%)	0.50	2
		37	17516	902 (88%)	0.43	2
DSPC: copolymer 1	7:3	25	7970	571	0.51	1
		37	11935	621 (88%)	0.33	2
DSPC: copolymer 1	5:5	25	5580	469 (83%)	0.52	2
		37	5980	687(54%)	0.50	3
DSPC: copolymer 2	9:1	25	15935	727 (89%)	0.49	2
		37	15484	1000 (87%)	0.49	2
DSPC: copolymer 2	7:3	25	12548	1867 (71%)	0.53	2
		37	7730	1400 (84%)	0.56	2
DSPC: copolymer 2	5:5	25	5730	1498 (61%)	0.50	2
		37	5680	902 (88%)	0.54	2
DSPC:DOPC:copolymer 1	9:1	25	16097	1953 (78%)	0.52	2
		37	13806	9812 (48%)	0.50	2
DSPC:DOPC:copolymer 2	9:1	25	15968	1181 (84%)	0.49	2
		37	16226	1122	0.52	2

* The parenthesis refers to systems with more than one peak and corresponds to the intensity weight of the main peak.

Table S4. Stability study of DSPC and DSPC:DOPC (9:1 weight ratio) hybrid systems incorporating stimuli-responsive copolymers P(OEGMA-co-DIPAEMA)-1 and -2.

Sample	w/w	t(days)	I (kHz)	R_h (nm) *	PDI
DSPC: copolymer 1	9:1	7	6400	85 (53%), 721 (47%)	0.46
		14	6860	86 (45%), 765 (55%)	0.49
		28	13290	275	0.43
DSPC: copolymer 1	7:3	7	6510	110 (35%), 516 (65%)	0.50
		14	8000	127 (56%), 887 (44%)	0.47
		28	6530	112 (40%), 927 (60%)	0.51
DSPC: copolymer 1	5:5	7	9100	175	0.48
		14	5180	188	0.49
		28	4730	95 (30%), 401 (70%)	0.39
DSPC: copolymer 2	9:1	7	5050	205	0.47
		14	6140	107	0.44
		28	7050	150	0.45
DSPC: copolymer 2	7:3	7	4650	82 (62%), 648 (38%)	0.44
		14	5030	79 (56%), 613 (44%)	0.45
		28	6170	100 (64%), 854 (36%)	0.45
DSPC: copolymer 2	5:5	7	2952	112 (93%)	0.38
		14	2797	96 (94%)	0.37
		28	3106	23 (11%), 109 (89%)	0.39
DSPC:DOPC:copolymer 1	9:1	7	8790	151 (44%), 1610 (51%)	0.49
		14	11548	142 (48%), 2076 (50%)	0.48
		28	12484	177 (46%), 2785 (52%)	0.48
DSPC:DOPC:copolymer 2	9:1	7	5430	102 (60%), 1431 (37%)	0.47
		14	5630	41 (19%), 194 (80%)	0.45
		28	7440	106 (41%), 337 (57%)	0.47

* The parenthesis refers to systems with more than one peak and corresponds to the intensity weight of each peak or the main peak when it is more than 90%.

Table S5. Thermodynamic evaluation of lipid/copolymer colloidal dispersions in different environments (aqueous and pH 4.5) as measured by microcalorimetry (mDSC) and high-resolution ultrasound spectroscopy (HR-US).

Sample	w/w	Medium	mDSC		HR-US	
					(sound speed)	(attenuation)
			Temperature (°C)	Enthalpy (J/g)	Transition temperature (°C)	
DSPC:1	9:1	water	54.21±0.03	0.192±0.014	56.60±0.31	56.10±0.23
		pH 4.5	55.07±0.06	0.214±0.039	56.41±0.22	55.20±0.09
DSPC:1	7:3	water	54.12±0.01	0.154±0.022	56.18±0.19	55.95±0.21
		pH 4.5	54.02±0.67	0.171±0.033	56.11±0.14	54.99±0.12
DSPC:1	5:5	water	53.93±0.04	0.134±0.17	56.08±0.33	55.58±0.18
		pH 4.5	55.06±0.28	0.211±0.034	56.83±0.24	55.52±0.10
DSPC:2	9:1	water	54.03±0.01	0.239±0.026	56.22±0.22	55.66±0.20
		pH 4.5	54.62±0.88	0.208±0.039	56.53±0.32	55.57±0.06
DSPC:2	7:3	water	53.94±0.03	0.157±0.008	56.10±0.19	55.58±0.17
		pH 4.5	54.95±0.06	0.197±0.019	56.68±0.26	55.58±0.15
DSPC:2	5:5	water	53.84±0.05	0.132±0.023	56.27±0.23	55.60±0.28
		pH 4.5	54.65±0.69	0.189±0.021	56.26±0.11	55.34±0.31
DSPC:DOPC:1	9:1	water	53.09±0.12	0.220±0.011	55.12±0.36	54.44±0.39
		pH 4.5	*No data available*[1]			
DSPC:DOPC:2	9:1	water	51.95±0.34	0.175±0.009	54.13±0.29	53.61±0.33
		pH 4.5	*No data available*[1]			

[1]DSPC:DOPC hybrid systems were investigated only in water dilution medium due to their limited further utilization in pharmaceutical applications.

Table S6. Properties of hybrid DSPC:P(OEGMA$_{950}$-co-DIPAEMA)-2 systems incorporating MTX the day of their preparation at ambient temperature.

Sample	lipid to polymer w/w	I (kcps)	R_h (nm)	PDI	%EE
MTX-DSPC:2 9:1	9:1	2003	55	0.23	87
MTX-DSPC: 2 5:5	5:5	1058	60	0.24	100

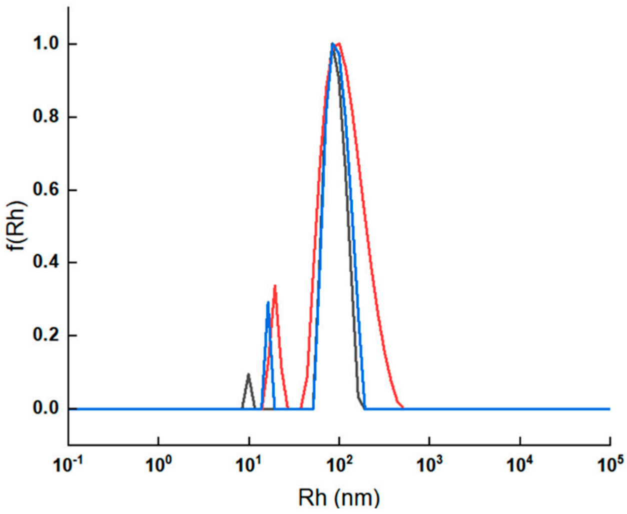

(a)

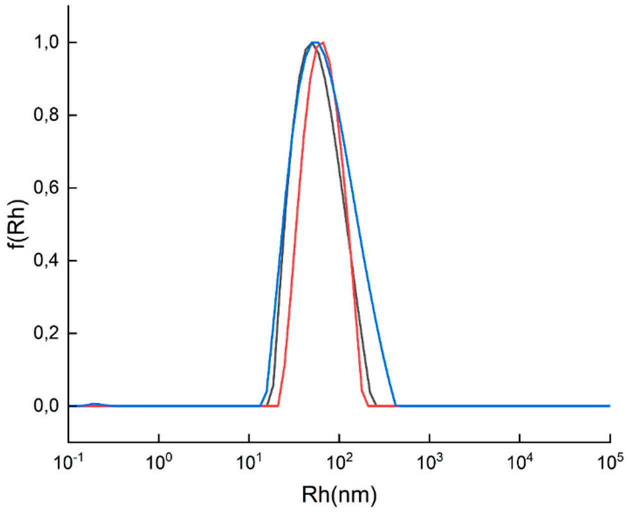

(b)

Figure S1. Size distributions via DLS of **(a)** empty and **(b)** MTX loaded DSPC:P(OEGMA-co-DIPAEMA)-2 hybrid system in a lipid to polymer weight ratio of 5:5 vs. time. Black line: t=0 days, red line: t=14 days, blue line: t=21 days.

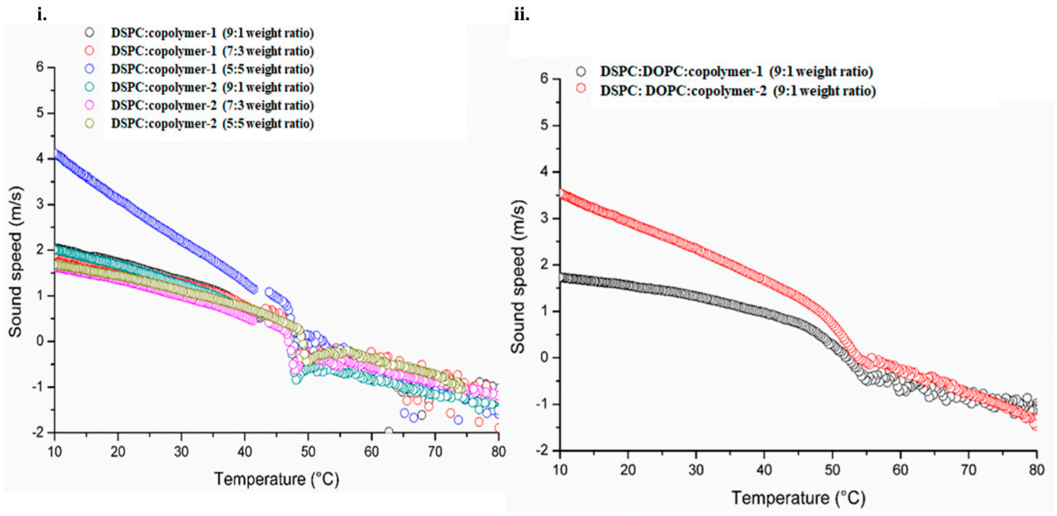

(a)

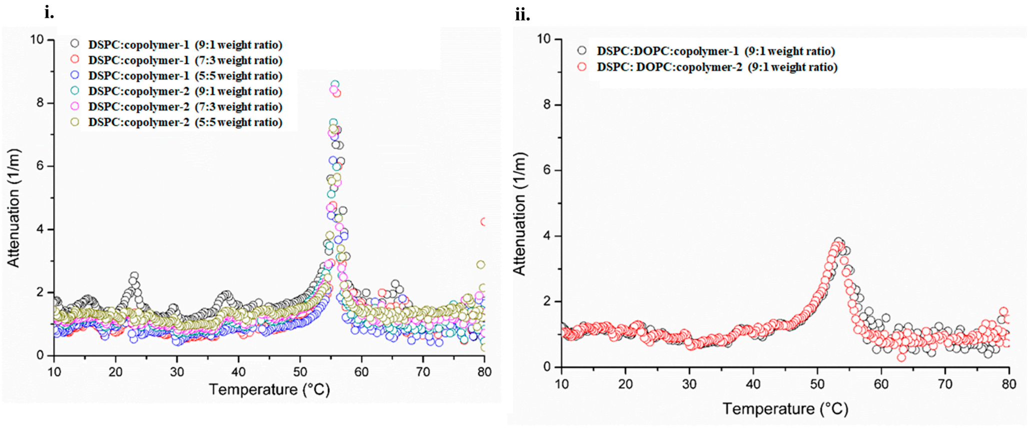

(b)

Figure S2. Charts from HR-US: **(a)** Sound speed; **(b)** Attenuation vs. temperature for the i. DSPC and ii. DSPC:DOPC (9:1 weight ratio) hybrid colloidal dispersions into aqueous medium.
